# Platelet-rich plasma as an effective biological therapy in early-stage knee osteoarthritis: One year follow up

**DOI:** 10.1051/sicotj/2021003

**Published:** 2021-03-01

**Authors:** Deepak Rai, Jyotsana Singh, Thimmappa Somashekharappa, Ajit Singh

**Affiliations:** 1 Senior Resident, Department of Orthopaedics, Trauma Center, BHU 221005 Varanasi India; 2 Junior Resident, Department of Pediatrics, JNMC, AMU 202002 Aligarh India; 3 Professor, Department of Orthopaedics, Rohilkhand Medical College 243006 Bareilly India; 4 Professor, Department of Orthopaedics, S.S. Hospital, BHU 221005 Varanasi India

**Keywords:** Osteoarthritis, Platelet rich plasma (PRP), WOMAC pain score

## Abstract

*Objective*: PRP is produced by centrifugation of whole blood containing highly concentrated platelets, associated growth factors, and other bioactive agents which has been shown to provide some symptomatic relief in early knee osteoarthritis (OA). The principal objective of our study was to evaluate the effectiveness and safety of standardized intra-articular injection of autologous PRP in early osteoarthritis knee. *Methods*: A total of 98 eligible symptomatic patients received two injections of standardized PRP 3 weeks apart. Clinical outcomes were evaluated using the VAS and Western Ontario and McMaster Universities Arthritis Index (WOMAC) questionnaire before treatment and at 6 weeks, 3 months, 6 months, and 1 year after treatment. Secondary objectives were safety (side effects), and the effect of PRP on the different grades of knee degeneration. *Results*: There was a statistically significant improvement in mean VAS and WOMAC scores at 6 weeks, 3 months, 6 months, and slight loss of improvement at 1 year follow-up. There was also a correlation between the degree of degeneration and improvement in the mean scores. The decrease in mean pain score is more in grades 1 and 2 (early OA) than in grade 3. The intraarticular injection is safe, with no major complications. *Conclusion*: PRP is a safe and effective biological regenerative therapy for early OA Knees. It provides a significant clinical improvement in patients with some loss of improvement with time. More studies will be needed to confirm our findings.

## Introduction

The etiology and pathogenesis of OA are complex and not quite clear and articular cartilage degradation and reactive formation of subchondral bone with mild synovitis are some of the common risk factors found in OA. Treatment of Knee OA is marred by the fact that the articular cartilage is aneural and avascular, which means a low regenerative capacity, and therefore a limited reparative potential of the joint [1]. The treatment is focused on means to mitigate patient symptoms and reduce the progression of the degenerative process as there is no definitive cure for OA [2]. Because of a lack of the effectiveness of conventional management options, current research focuses on targeting biological pathways that trigger the change in the homeostasis of joint and accelerate joint healing [3].

In recent years Platelet-rich plasma (PRP) is emerged as a promising treatment modality and classified as “Orthobiologics”. PRP enhances tissue recovery, by catalysing the body’s natural healing response and tissue repair process [3]. Platelet alpha-granules contain and release numerous growth factors such as platelet-derived growth factor (PDGF), vascular endothelial growth factor (VEGF), transforming growth factor-B (TGF-B), and hepatocyte growth factor (HGF) [4], which can potentially change the joint environment in OA. PRP promotes chondral remodelling and chondrocyte proliferation as it increases the synthesis of collagen II, prostaglandin (PG), and matrix molecules [5, 6]. In platelets microvesicles, different microRNAs [6, 7] involved in mesenchymal tissue regeneration [6, 8] are also present and microRNA-23b has been hypothesized to be strictly involved in the differentiation of MSC into chondrocytes [6–8].

PRP helps to create a highly favourable and balanced environment for angiogenesis by increasing hyaluronic acid (HA) secretion [9, 10], and at the same time, it decreases interleukin-1 (IL-1)-mediated increase of some matrix metalloproteinases (MMPs) [9, 10]. PRP has been used in the treatment of osteoarthritis knee and has shown promising clinic-radiological outcomes, both in comparison to pharmacological and non-pharmacological treatment modalities. This clinical study was designed to find the role of PRP in knee OA. The primary objective was a percentage reduction in mean pain scores at one year. Secondary objectives were safety (side effects), and the effect of PRP on the different grades of knee degeneration.

## Materials and methods

This was a prospective, longitudinal, non-randomized study done at a tertiary care university teaching hospital. It evaluated the effectiveness of a cycle of two intra-articular injections of autologous PRP in knee OA patients. Patients who were diagnosed as having OA Knee as per American College of Rheumatology criteria [7] and staged as per Ahlbäck radiological grading [11] were included in this study, based on *inclusion criteria*:

1. Patient having OA knee with a history of chronic pain.
2. Radiological findings of degenerative changes.
3. Ahlbäck grade 1, 2, and 3 knees without significant deformity.

*Exclusion criteria* were

1. OA secondary to inflammatory joint disease, metabolic diseases of the bone, haematological disease, severe cardiovascular condition, infections, or traumatic osteoarthritis or tense joint effusion.
2. Patient receiving immunosuppressants or anticoagulants.
3. Patients with platelet count less than 1,50,000/cu mm and Hb of less than 9 gm%.
4. Patients who had taken any intra-articular injections in preceding 3 months or an arthroscopic lavage in the last year.

After institutional ethics board clearance, patients giving their consent for the study were received injections as per standardized protocol. After enrolment out of 138 subjects, 36 had to be removed from the study because they met one or the other exclusion criteria.

### PRP preparation

We used a 20 cc syringe with 2 cc of anticoagulant and 18 cc venous blood was drawn and mixed well. Blood was injected into the “REMI” PRP centrifuge vial through the upper port. After 1st centrifugation in “REMI” PRP centrifuge, the height was adjusted to separate the boundary by pulling the knob up and down. Plasma and the RBC layer were blocked completely. After the 2nd centrifugation in “REMI” PRP centrifuge, the upper silicone lid was opened and the PRP was then extracted done by a pipette. A leucocyte filter was then used to filter off the leucocytes, PRP activation was done immediately before injection by adding 10% calcium chloride. PRP was injected out with the help of a 10 mL syringe. Finally, the platelet concentrate was mixed and drawn. The Platelet-rich plasma was divided into 2 units in disposable syringes. One unit was sent for analysis of platelet concentration and quality test and the second part was used for the first dose of intra-articular infiltration in patients within two hours of preparation.

The first injection was given on the same day, under aseptic conditions. 10 mL of PRP was injected into the knee joint through the anterolateral approach with a 22-gauge needle. After the injection, the patient was encouraged to move the knee a few times to allow the Platelet-rich plasma to spread in the knee joint after that knee was kept in extension for 20 min. After injection, some patients developed complications like sweating, dizziness, and nausea, were observed and discharged when fully recovered. All patients were followed up at 6 weeks, 3 months 6 months, and at 1 year. The quality of life was assessed using Western Ontario and McMaster Universities Arthritis Index (WOMAC) scoring and Visual Analog Scale (VAS) for pain, before starting the treatment and then at 6 weeks, 3 months, 6 months, and 1 year of treatment.

Data analysis was done with the SPSS 26. The descriptive analysis (e.g., mean and standard deviation) was done for normally distributed parameters and their means were compared using the analysis of variance (ANOVA) tests. Within the groups, the data on pre and post levels were compared using the Student *t*-test. Data of subsequent follow-ups were analysed using repeated-measures ANOVA which was followed by post hoc tests. *P*-value of less than 0.05 was taken as significant in all the tests.

## Results

In the present study, 102 patients with knee OA received PRP injections. Out of these 102 patients, 4 patients were lost to follow up. Out of 98 patients who were finally analysed, 62 were female and 36 were male, with a mean age of 60.33 ± 4.21 (range 52–70 years). The majority of patients were Ahlbäck grade 2 (44.8%), followed by grade 3 (28.5%) and the rest were grade 1 (26.5%). Only 9 patients developed complications like syncope, dizziness, headache, and sweating, which were of short duration, lasting for 20–30 min. No serious complications related to the injections were observed during the follow-up period.

1. WOMAC pain score: Pre-treatment WOMAC pain score was 15.51 ± 1.459 and there was a significant decrease in mean pain score at each subsequent follow-up. There was a slight increase in mean pain score at 6 months and 1 year of follow-up, however, the mean pain was still significantly lower than that of baseline (Table 1). Follow-up comparisons by repeated measure ANOVA indicated a significant decrease in WOMAC pain score, Wilks’ lambda = 0.016 *F*(4, 45) = 678.908, *P* < 0.01. Follow-up comparison indicates there was a correlation between Ahlbäck grade and decrease mean pain score, with grade 1 knee having lower mean pain than grade 2 knee and grade 2 having lower mean pain score than grade 3 (Figure 1).
  **Figure 1:**
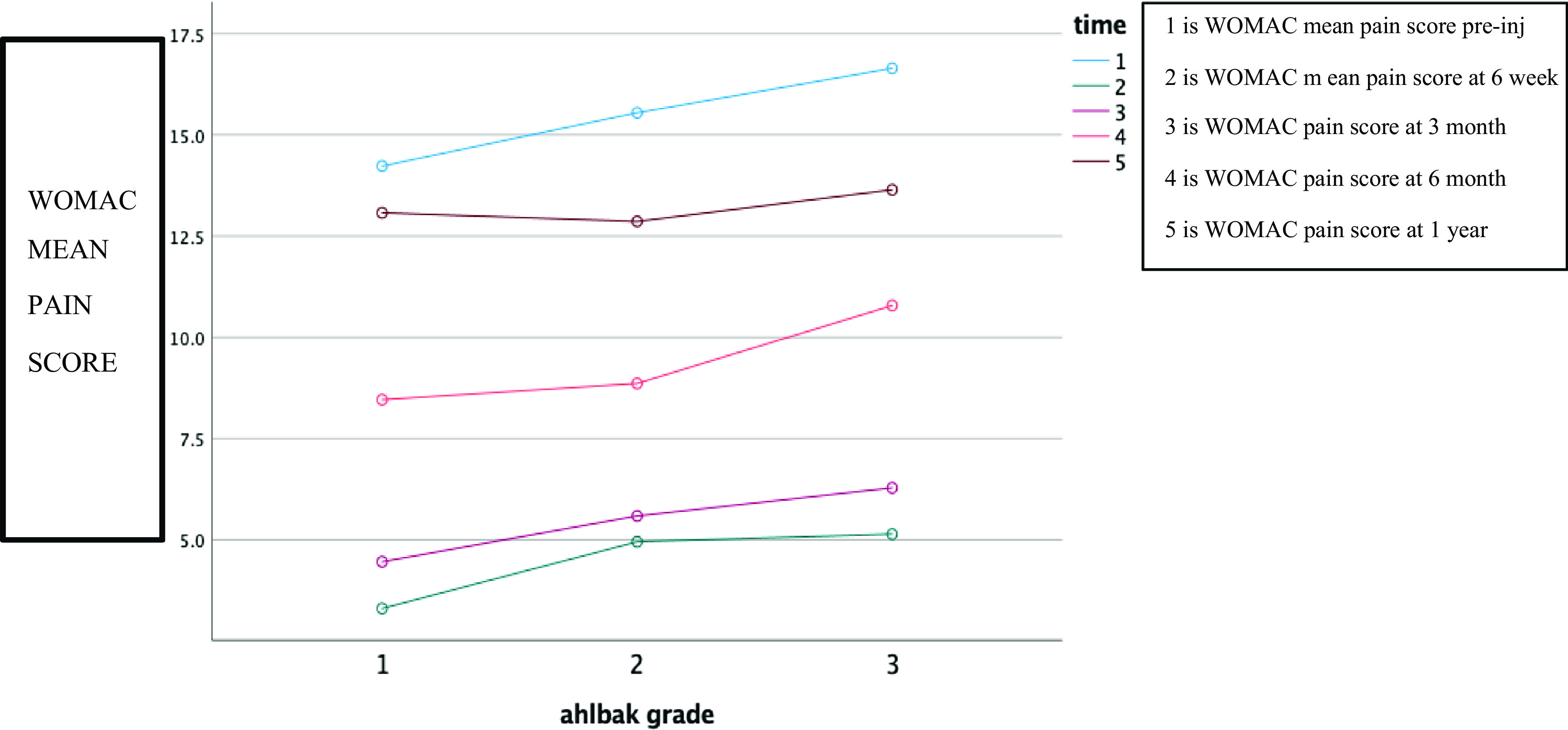
Influence of knee degeneration on WOMAC scores. The overall best improvement after six months and 1 year was observed in Ahlbäck I sub-group and the overall least improvement in Ahlbäck III sub-group. This shows a statistically significant improvement (*p* < 0.05) in all sub-groups after PRP injection.
  **Table 1:** Percentage change in WOMAC and VAS scores at 6 weeks, 3 months, 6 months, and at one year after PRP injection compared to pre-injection levels. On applying paired *t*-test, *P*-value <.001, so there is a significant change in mean VAS and WOMAC function score post-injection.

Clinical parameter	Time				
	PREINJ	6 weeks	3 months	6 months	1 yr
WOMAC pain					
(A) Mean pain	15.51	4.57	5.49	9.31	13.14
(B) % change		70.6%	64.7%	40%	15.3%
(C) *P*-value	On applying paired *t*-test *P*-value < .001, so there is a significant change in mean pain score post-PRP.				
WOMAC stiffness					
(A) Mean pain	5.51	2.57	3.12	3.80	4.96
(B) % change		63.4%	53.4%	31.15%	10%
(C) *P*-value	On applying paired *t*-test *P*-value < .001, so there is a significant change in mean WOMAC stiffness score.				
WOMAC function					
(A) Mean pain	57.96	16.63	17.84	37.43	54.27
(B) % change		71%	69.3%	35.4%	6.4%
(C) *P*-value	On applying paired *t*-test, *P*-value < .001, so there is a significant change in mean WOMAC function score post-injection.				
VAS score					
(A) Mean pain	6.06	2.24	2.49	3.14	4.80
(B) % change		64%	59%	48.2%	20.8%
(C) *P*-value	On applying paired *t*-test *P*-value < .001, so there is a significant change in mean VAS score.
2. VAS Pain Scores: Pre-treatment VAS pain score was 6.06 ± 1.36 and there was a significant decrease in mean pain score at subsequent follow-up. There was a slight rise in mean pain score at 6 months and 1 year of follow-up, however, the mean VAS pain was still lesser than that of baseline (Table 1). Follow-up comparisons by repeated measure ANOVA indicated a significant decrease in VAS pain score, Wilks’ lambda = 0.046 *F*(4, 43) = 223.265, *P* < 0.01. A descriptive analysis of the means on applying repeated measured ANOVA, showed an overall significant decrease in VAS pain score in all Ahlbäck grades as compared to the pre-treatment scores. Best results after 6 weeks, 3 months, and 6 months in Ahlbäck I subgroup, and the overall least improvement in Ahlbäck III subgroup (Figure 2).
  **Figure 2:**
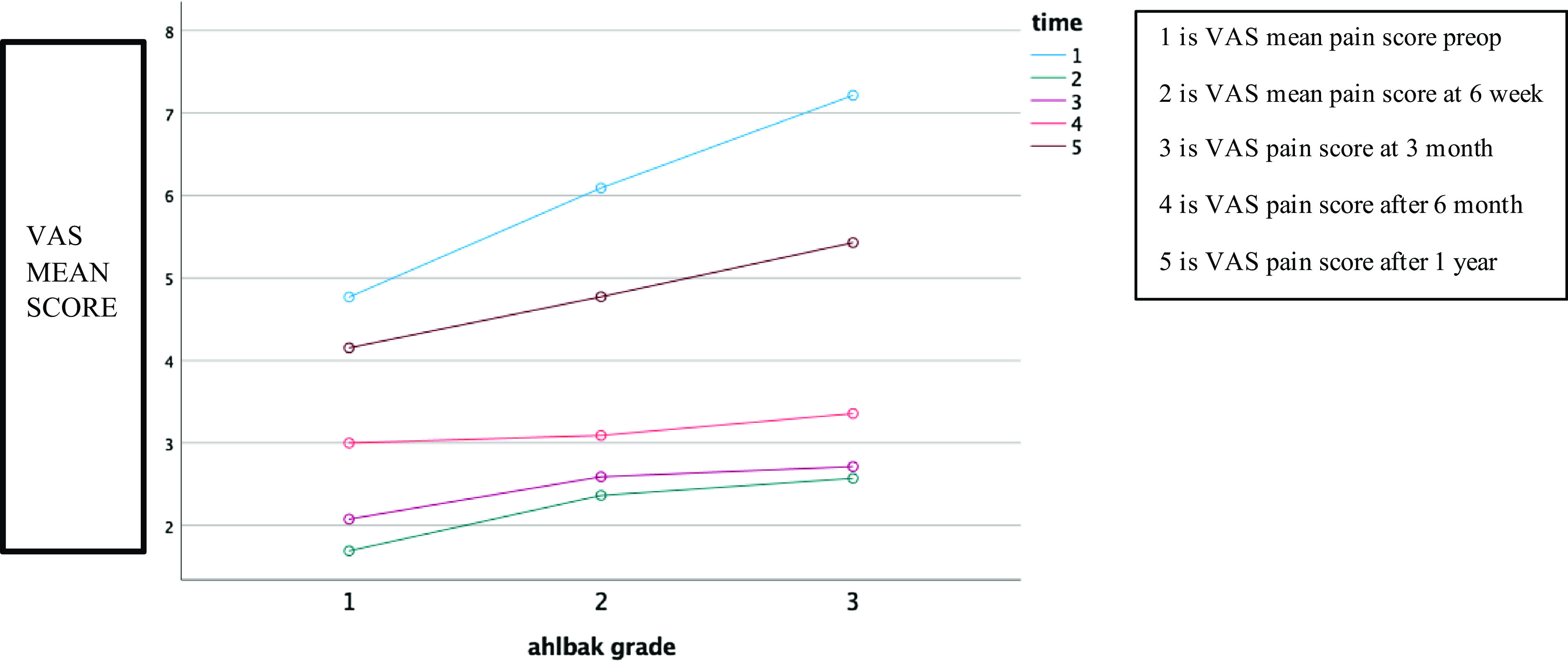
Effect of knee degeneration grade on VAS. The overall best improvement after 6 months and 1 year was observed in Ahlbäck I sub-group and the overall least improvement in Ahlbäck III sub-group. This shows a statistically significant improvement (*p* < 0.05) in all sub-groups after PRP injection.
3. The pattern of other secondary WOMAC parameters like stiffness and physical function was similar to pain pattern (Table 1).
4. There was no correlation of WOMAC parameters with age, sex, weight, or height.

## Discussion

Several pharmacological and non-pharmacological treatment options have been suggested for the management of OA Knees. PRP has shown a very good promise as an agent of tissue repair and regeneration. Most of the studies involving the use of PRP point towards some improvement in pain and function but lack proper documentation and analysis [12–15].

Looking at the primary outcome measure i.e., pain, we observed that there was a significant reduction in mean pain score. Although there was albeit a small rise in mean pain scores at the final 1-year follow-up even then, the final pain scores were still significantly lower than the baseline pain scores. Kon et al. [16] in their study also showed similar results, and they found a slight decrease of knee score (IKDC) from 2 months to 6 months, but that was not significant. Patel et al. [17] also noted significant improvement in all WOMAC scores within 2 to 3 weeks which lasted up to 6 months (the final follow-up), with some worsening at 6 months. Bottegoni et al. [18] in their study observed, a statistically significant improvement of IKDC, KOOS, and VAS at a follow-up of two months, but there was a statistically significant worsening between two months to the six-month follow-up period.

In our study improvement was shown to continue till 1 year follow-up and this may be attributed to the technique used by us. Despite showing promising results in OA, PRP has not gained acceptance and confidence as a valid treatment option. This is also because of the lack of our knowledge regarding plasma rich platelet segregation techniques and current literature lacks standardization of study protocol and outcome analysis. PRP concentrate, in terms of platelet count and leucocyte concentration, is affected by the number of spins (double vs. single), centrifugal forces, and time. We have used double spin and type 4B leucocytes depleted and activated PRP containing about 4× platelet concentration above baseline (Sports Medicine Platelet-Rich Plasma classification system by Mishra et al. [19]). Leucocytes can be proinflammatory within the joint and be harmful [17, 19]. We used fresh PRP preactivated by CaCl_2_ before injection which has been found to be better than thawed PRP [17, 19]. We believe that our slightly better results are due to standardized technique which has gradually evolved and should be the preferred standard technique for further clinical use.

In this study, we found a definite correlation between Ahlbäck grading and a decrease in mean pain and other WOMAC scores and VAS score. The decrease in mean pain score is more in grades 1 and 2 than grade 3 and on subsequent follow up slight worsening occurs, which is more in grade 3. Such findings were also reported in a study by Bottegoni et al. [18] in which statistically significant improvement in terms of mean and at 2- and 6-month evaluations was observed in Ahlbäck I–II knee OA and overall poor results in terms of means were found in Ahlbäck III sub-group and patients aged 80 years or more. The above improvement in symptoms can be explained by modulating the joint environment and affecting the concentration of cytokinin and inflammatory cascades. PRP does not cause structural changes in the joint as the effect is for a short to medium duration only. But in this duration patients can be put on active physical therapy to sustain the improvement to avoid replacement surgery.

Intra-articular infiltration of autologous PRP was well tolerated by all patients included in the study. These findings are also consistent with the results of Patel et al. [17] who treated degenerative cartilage lesions of the knee with intra-articular PRP in 78 patients. They postulated that adverse effects are mainly due to more quantity of platelet in PRP or because of the use of CaCl_2_. Glynn et al. [20] in their study observed that PRP has minimal associated adverse events and may have beneficial effects in terms of pain, health utility, patient satisfaction, and goal-orientated outcomes.

### Limitations

There are few limitations in our study including lack of randomization and placebo control groups and a relatively short follow-up. An investigation like histological, biochemical analysis, and radiographic investigation like MRI can be used for evaluating cartilage regeneration in future studies that have not been used in the current study.

## Conclusion

This study shows that intraarticular infiltration of PRP in the OA knee is tolerable, very safe while being effective. Future studies with a larger sample size and a longer follow-up will be required to confirm the role of PRP as an effective biological therapy in OA Knee.

## Conflict of interest

Author declared no conflict of interests.
